# Solitary pulmonary metastasis from a gingival cancer of 36 years ago: A case report

**DOI:** 10.1016/j.ijscr.2018.11.053

**Published:** 2018-11-24

**Authors:** Atsushi Kamigaichi, Kei Shimizu, Yoshihiro Miki, Katsura Hakamada, Yoshiro Otsuki, Toru Nakamura

**Affiliations:** a Departments of General Thoracic Surgery, Seirei Hamamatsu General Hospital, Japan; b Departments of Respiratory Medicine, Seirei Hamamatsu General Hospital, Japan; c Departments of Otolaryngology, Seirei Hamamatsu General Hospital, Japan; d Departments of Pathology, Seirei Hamamatsu General Hospital, Japan

**Keywords:** CT, computed tomography, SqCC, squamous cell carcinoma, ACC, adenoid cystic carcinoma, HNCs, head and neck cancers, 18-FDG-PET, 18-fluorodeoxyglucose positron emission tomography, DFI, disease free interval, Solitary pulmonary nodule, Adenoid cystic carcinoma, Head and neck cancer metastasis, Case report

## Abstract

**Introduction:**

Unlike a squamous cell carcinoma (SqCC), an adenoid cystic carcinoma (ACC) is an uncommon histology of all head and neck cancers. The clinical significance of a pulmonary metastasectomy differs greatly between these two types of cancers. A solitary pulmonary nodule in a patient with a history of both these different malignancies is a diagnostic and therapeutic challenge.

**Presentation of case:**

An 81-year-old woman presented with a right lung nodule detected by chest computed tomography (CT) one year after a tongue SqCC surgery. She had a remote history of gingival cancer 36 years prior, which was not assessed in detail during the initial work up. We suspected that the nodule was a primary lung cancer and she underwent a right basal segmentectomy. Histology revealed an ACC, which was proven to be a metastasis from the gingival cancer.

**Discussion:**

An ACC is an uncommon type of cancer arises mainly in the salivary glands. It is characterized by a long-term behavior and the histology of ACC is a favorable prognostic factor. With its favorable histology and disease free interval of over 30 years, a better outcome would be expected than that of a primary lung tumor or a metastasis from the tongue SqCC. Furthermore, we could have made a differential diagnosis of a metastatic ACC before the surgery with more careful research for a previous history of a gingival cancer.

**Conclusion:**

Our case emphasized that a precise history taking of any malignancy, even if that of more than 30 years prior, is crucial.

## Highlights

• An adenoid cystic carcinoma is characterized by a long-term behavior and late recurrences.
• The recurrences of ACC after more than 30 years have not been reported.
• The precise preoperative diagnosis was very difficult due to a history of tongue cancer one year previously in the present case.
• We should never overlook a history of any malignancy even if it is over 30 years prior.

## 1 Introduction

A pulmonary metastasectomy is a feasible treatment option especially in colorectal cancer patients. In contrast, its clinical significance in head and neck cancers (HNCs) much differs among each primary site. The most common histology of all HNCs is a squamous cell carcinoma (SqCC), whereas an adenoid cystic carcinoma (ACC) is uncommon and accounts for only 3–5% of these cancers [1]. Although both cancers could metastasize to the lungs, the clinical significance of a pulmonary metastasectomy differs greatly because of its distinct survival probability. Therefore, a solitary pulmonary nodule in a patient with a history of both these different malignancies is a diagnostic and therapeutic challenge. We herein report a case of a metastatic lung tumor not from a tongue SqCC of one year prior, but from a gingival ACC of 36 years prior. This paper has been dictated fulfilling by the SCARE criteria [2].

## 2 Presentation of case

**Fig. 1:**
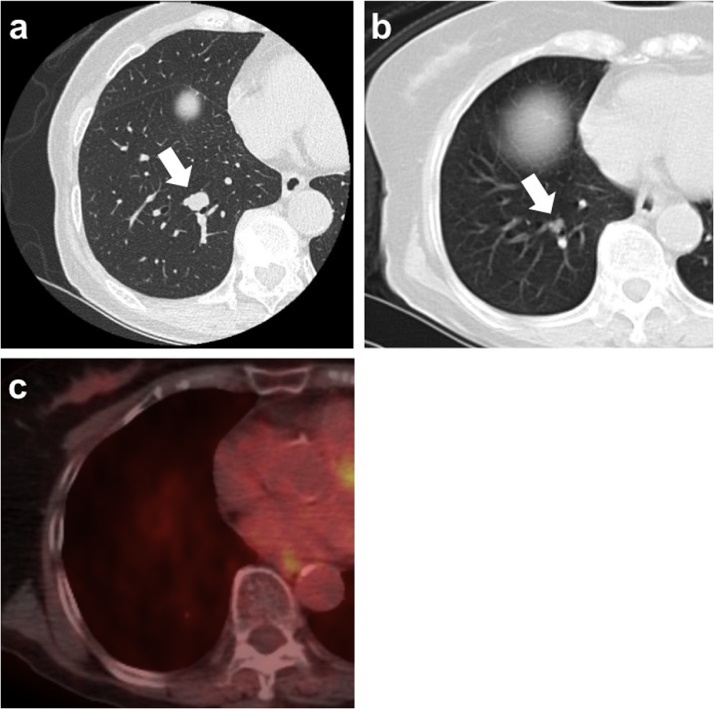
(a) Computed tomography of the chest showing a lung nodule measuring 12 × 10 mm in the right basal segment (arrow). (b) Computed tomography one year prior showing a lung nodule measuring 7 × 6 mm (arrow). (c) Positron emission tomography showing no uptake of 18-fluorodeoxyglucose into the lung nodule.

An 81-year-old woman with a history of tongue cancer surgically treated one year previously presented with a right lung nodule detected by chest computed tomography (CT) as a postoperative follow up. Regarding a tongue cancer, a curative resection was performed and the pathological staging was StageⅡ (T2N0M0). She had no smoking history or drinking habit and was without any symptoms. Her human papillomavirus infection status was not assessed. She had a remote history of a right lower gingival cancer 36 years prior (at the age of 45) followed by a cervical lymph node metastasis 5 years later. However, we did not assess the medical history about gingival cancer in detail during the initial work up. The chest CT revealed a solitary pulmonary nodule measuring 12 × 10 mm with a clear margin in the right lower lobe that had grown from 7 × 6 mm at the time of the initial diagnosis of the tongue cancer one year prior (Fig. 1a, b). There was no uptake on the 18-fluorodeoxyglucose positron emission tomography (18-FDG-PET) (Fig. 1c). We suspected the nodule was a primary lung cancer rather than a metastasis from the tongue cancer because solitary pulmonary nodule in patients with a history of HNC is more likely to be a primary cancer than a metastasis [3]. Furthermore, we did not even suspect the possibility of a metastasis from the gingival cancer at all only because it was “36” years prior.

　The transbronchial biopsy appeared to be undiagnostic because of the lack of any bronchial involvement of the lesion. Further, neither a percutaneous needle biopsy nor wedge resection of the lung was appropriate because the lesion was located close to the hilum and there was the risk of bleeding due to those interventions. Therefore, an anatomical pulmonary resection was recommended and we chose a segmentectomy as a passive sublober resection because of old age and a short time from tongue cancer surgery.

Because a poor prognosis was expected even after a curative resection in the case of a metastatic SqCC [4], we carefully obtained informed consent. She underwent a right basal segmentectomy with a hilar lymph node dissection. The postoperative course was uneventful and she was discharged on the 6th postoperative day.

**Fig. 2:**
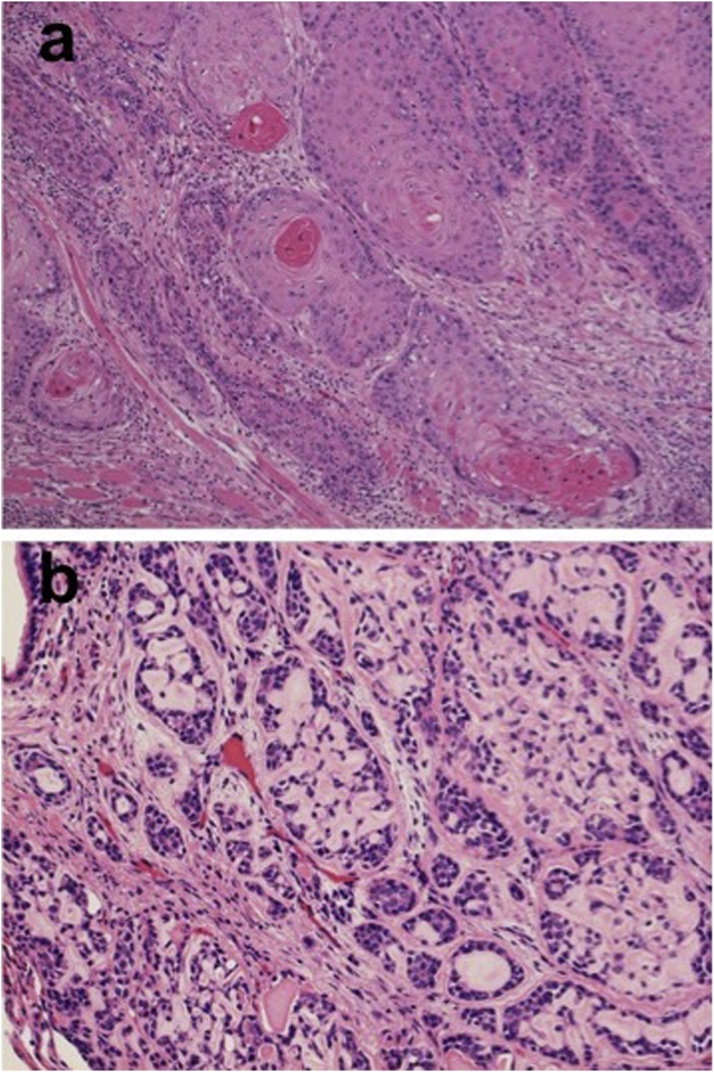
(a) Histologically the tongue lesion shows an invasive squamous cell carcinoma with dyskeratosis (hematoxylin and eosin, X150). (b) Histologically the right lung nodule exhibits tubular and cribriform patterns containing an amorphous eosinophilic material (hematoxylin and eosin, X150).

**Fig. 3:**
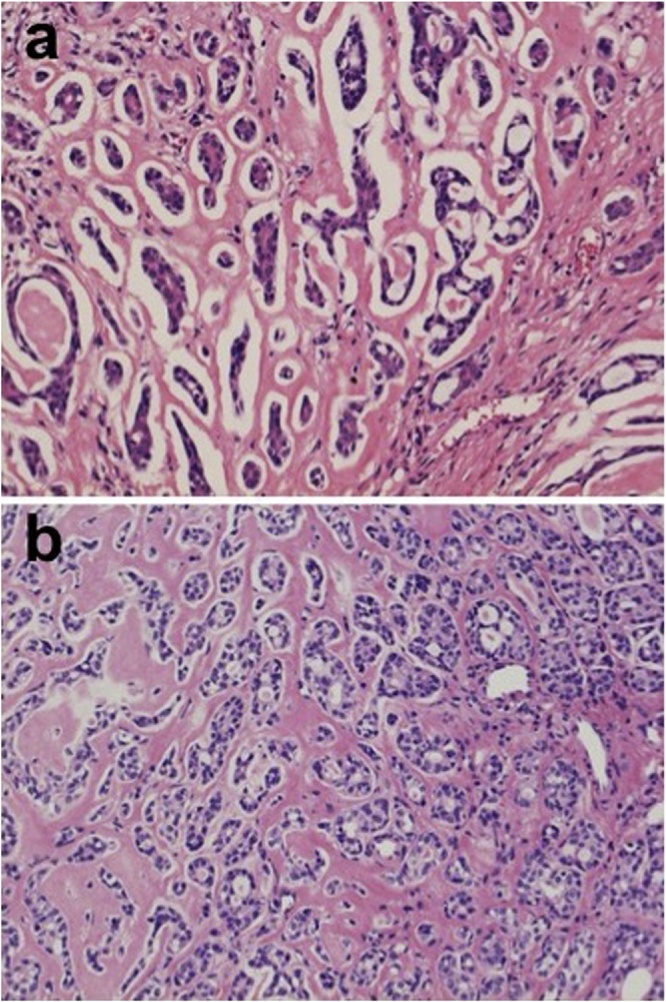
Histologically both a gingival cancer from 36 years prior (a) and a cervical metastasis 31 years prior (b) revealed tubular and cribriform patterns with amorphous eosinophilic material (hematoxylin and eosin, X150).

The histological findings of the surgical specimen revealed a cribriform pattern. It was a typical finding of an ACC which was different from the histology of the tongue SqCC one year prior (Fig. 2a, b). We retrospectively reviewed the histology of the gingival cancer resected 36 years previously, which revealed a cribriform pattern just the same as that of the lung nodule (Fig. 3a). A metastatic lymph node, dissected 31 years previously also revealed the same histology as the ACC (Fig. 3b). With these histological findings and past history, we diagnosed the lung nodule as a metastasis from the gingival cancer. She is currently disease free at 2 year after the pulmonary metastasectomy.

## 3 Discussion

The clinical significance of a pulmonary metastasectomy differs among the histologies of each HNC. The histology of ACC is a favorable prognostic factor and patients with an ACC have a 5-year survival rate of 84% after a pulmonary metastasectomy [5,6]. However, a pulmonary metastasectomy is not even indicated for a tongue SqCC, which is the most common histology of all HNCs, due to a quite worse prognosis with a 3-year survival rate of 7% [4]. In the present case, we suspected the solitary pulmonary nodule was a primary lung cancer, therefore the histological examination was essential. Due to its location close to the hilum, we chose a surgical resection. We considered that although surgery could have been curative in the case of a primary lung cancer, it might have been only diagnostic in the case of a metastasis from a tongue SqCC. However, it turned out to be a metastatic ACC from the gingiva, which could have a longer life expectancy and we had never considered even its possibility before the surgery.

An ACC is an uncommon type of cancer, accounting for only 3–5% of all HNCs, and arises mainly in the major and minor salivary glands [1]. It is characterized by a long-term behavior with a 20-year survival rate of 28% and late recurrences as in our case [7]. Lung is a common site of distant metastasis from a salivary gland ACC and pulmonary metastasectomy is considered to be one of the feasible therapeutic options [8,9].

In this case, we had to consider a primary ACC of the lung as a differential diagnosis. A primary ACC of the lung is a particularly rare disease, accounting for only 0.04% of all primary lung cancers, and more likely to be located in the trachea and rather central bronchi in many cases [10,11]. Considering the rarity of primary ACC both in the lung and head and neck area, it is quite unlikely that such rare primary tumors develop in the same individual separately. So we diagnosed the lung nodule in our case as a metastasis from gingival ACC which had previously developed a lymph node metastasis in the past.

Many previous reports have showed a long disease free interval (DFI) between initial treatment of the primary ACC and the development of lung metastasis. However, there is no case which DFI is over 30 years as in our case [8,9,[12], [13], [14], [15], [16]]. In general, it is considered that a longer DFI is a better prognostic factor in metastatic lung tumors and the same is true of metastatic pulmonary tumor from salivary grand ACC [6,8,9,17]. Our case could have a good prognosis because of the longer DFI of over 30 years and a favorable histology as ACC. Although FDG-PET provides more accurate information for salivary gland malignancies, the standardized uptake value in ACCs is quite low [18]. This is consistent with the finding in our case, but was not diagnostic because we did not consider the possibility of an ACC before the surgery just because it was over 30 years prior. With more careful research for a previous history of a gingival cancer and the consideration of the radiological findings, we could have made a differential diagnosis of a metastatic ACC before the surgery.

## 4 Conclusion

We report a case of solitary pulmonary metastasis who had two different cancers with distinct significance of metastatsectomy. As a result, the right lung nodule was proven to be a tumor with a favorable prognosis. In summary, we should never overlook a history of any malignancy even if it is over 30 years prior, in order to an accurate therapeutic strategy and not to miss the chance of a cure of the disease.

## Conflicts of interest

The authors declare no conflicts of interest.

## Funding

The authors declare that this study was not funded externally.

## Ethical approval

A case report is exempt from ethnical approval in our institution.

## Consent

Written informed consent was obtained from the patient for the publication of this case report and any accompanying images. A copy of the written consent is available for review by the Editor-in-Chief of this journal.

## Author contribution

AK and TN drafted the manuscript. AK, YM, KH and TN contributed to patient care. AK, KS and TN performed the literature search. YO performed histopathological examination and diagnosis. AK, KS, YM, KH and TN participated in the critical revision of the manuscript. All authors have read and approved the final manuscript.

## Registration of research studies

This is a case report.

## Guarantor

Atsushi Kamigaichi and Toru Nakamura.

## Provenance and peer review

Not commissioned, externally peer reviewed.
